# Outcomes of Preprocedural Pulmonary Hypertension on All-Cause and Cardiac Mortality in Patients Undergoing Transcatheter Aortic Valve Implantation: A Systematic Review

**DOI:** 10.7759/cureus.34300

**Published:** 2023-01-28

**Authors:** Aditya Desai, Darshi M Desai, Aneeque Jamil, Denise Csendes, Sai Dheeraj Gutlapalli, Keerthana Prakash, Kiran Maee Swarnakari, Meena Bai, Mohana Priya Manoharan, Rabab Raja, Safeera Khan

**Affiliations:** 1 Internal Medicine Clinical Research, California Institute of Behavioral Neurosciences and Psychology, Fairfield, USA; 2 Internal Medicine, University of California Riverside School of Medicine, Riverside, USA; 3 Internal Medicine, California Institute of Behavioral Neurosciences and Psychology, Fairfield, USA; 4 Medicine, California Institute of Behavioral Neurosciences and Psychology, Fairfield, USA

**Keywords:** pulmonary hypertension, pulmonary artery systolic pressure, morbidity and mortality, critical aortic stenosis, trans-catheter aortic valve replacement

## Abstract

Patients with symptomatic aortic stenosis (AS) commonly have an associated finding of pulmonary hypertension (PH), and it has been previously shown to have increased morbidity and mortality following surgical aortic valve repair (SAVR) as well as transcatheter aortic valve implantation (TAVI). There are no guidelines stating the cut-off point for PH at which the patient can safely undergo TAVI with benefits outweighing the risks. This is partly due to the lack of uniformity in the PH definition used in various studies. This systematic review sought to study the effect of preprocedural pulmonary hypertension on early and late all-cause and cardiac mortality in patients undergoing TAVI. We performed a systematic review of studies comparing patients with AS undergoing TAVI having PH. The review was undertaken as per Preferred Reporting Items for Systematic Reviews and Meta-Analyses (PRISMA) guidelines. Articles were identified from PubMed, Pubmed Central (PMC), Cochrane, and Medline on January 10, 2022, for literature published until January 10, 2022. MeSH strategy was used on PubMed to search the literature, and filters were applied to search only Observational Studies, randomized controlled trials (RCT), and meta-analysis. A total of 170 unique articles were identified and screened. Of the 33 full-text articles that were reviewed, 18 articles, including duplicates, were excluded. Fifteen articles fulfilled the selection criteria and were included in this review. The study design included two meta-analyses, one randomized control trial, one prospective cohort study, and 11 retrospective cohort studies. The studies involved a total of approximately 30,000 patients. The observational studies in our review were of good to fair quality, the RCT had a low to moderate bias, and the meta-analysis was of moderate quality. Baseline PH and persistence of PH post-TAVI are strongly associated with all-cause and cardiac mortality. Few studies have shown that a decrease in post-TAVI PH carries mortality benefits. Therefore, efforts should be made to identify mechanisms of persistent PH post-TAVI and whether interventions to reduce PH pre-TAVI will have any clinical implications or not by conducting RCT.

## Introduction and background

Patients with symptomatic aortic stenosis (AS) commonly have an associated finding of pulmonary hypertension (PH), and it has been previously shown to have increased morbidity and mortality following surgical aortic valve repair (SAVR) as well as transcatheter aortic valve implantation (TAVI) [1-4]. TAVI has been a safe and effective line of treatment for AS and is gaining more attention than SAVR. But because PH carries a higher perioperative risk for any procedure, its benefits versus risk in this population warrants investigation.

PH can be further classified into three main types: pre-capillary PH (Pc-PH), isolated post-capillary PH (Ipc-PH), and combined pre-capillary and post-capillary PH (Cpc-PH). Patients with AS having PH can fall into any of the three categories, but Ipc-PH is the most common subtype [5-7]. Ipc-PH can be found in up to 75% of patients with symptomatic AS. It is due to left-sided heart disease.

In the past few years, a new concept of AS-related cardiac injury has been introduced, which is thought to be due to the downstream effects on the left ventricle, left atrium, pulmonary vasculature, and ultimately the right side of the heart [8,9]. Ipc-PH and Cpc-PH represent an advanced stage of the cardiopulmonary process, thought to be due to the functional and structural abnormalities of the aortic valve causing injury to the downstream part of the cardiopulmonary system [10]. A minority of the patients with AS may suffer from Pc-PH or, Ipc-PH, which is not related to AS but attributable to other comorbidities such as chronic lung disease and other structural heart diseases [11].

A limited number of studies have analyzed post-TAVI outcomes among different entities of PH [12-14]. In addition, the amount of data on TAVI-induced hemodynamic changes in PH among subtypes of PH is scarce [15-18]. Fewer studies have reported PH as an independent risk factor post-TAVI and a preprocedural risk factor for TAVI. However, the drawback is that these studies lack a uniform definition of PH [6,18-21]. Although it is known that PH is a risk factor and an important determinant of surgical risk, it is not incorporated into the commonly used Society of thoracic surgeon score (STS score), and only severe PH with pulmonary artery systolic pressure (PASP > 60 mm Hg) is considered for European System for Cardiac Operative Risk Evaluation (EuroSCORE).

The evidence for short- and long-term mortality associated with PH after TAVI is inconclusive [11,12,15,17,21]. Few studies have shown the mortality benefits of improving PH post-TAVI [15,18]. No guidelines state the cut-off point for PH at which the patients can safely undergo TAVI, with benefits outweighing the risks. Therefore, this systematic review sought to study the effect of preprocedural PH on early and late all-cause and cardiac mortality in patients undergoing TAVI.

## Review

Methods

This systematic review was conducted according to Preferred Reporting Items for Systematic Reviews and Meta-Analyses (PRISMA) guidelines [22]. AD and DD performed a literature search in several databases, including PubMed, PubMed Central (PMC), ScienceDirect, Cochrane, and Medline, on January 10, 2022, for literature published between January 1, 2015, and January 10, 2022. MeSH strategy was used on PubMed to search the literature, and filters were applied to search only Observational Studies, Randomized Controlled Trials (RCT), and Meta-Analysis. The following MeSH keywords were used ("Aortic Valve Stenosis") AND ("Transcatheter Aortic Valve Replacement") AND ("Hypertension, Pulmonary"). We applied filters to narrow search results to human subjects, English-only, and by study type to exclude conference abstracts, case reports, comments, editorials, essays, letters, reviews, and unpublished studies. Duplicates were removed. Any additional articles identified by other means were added. We included studies with TAVI, defining the mode of diagnosing PH, papers published in the English language, papers relevant to the question, and papers focusing on outcomes of TAVI with a history of PH. We excluded studies discussing SAVR, papers on PH and AS but no mention of TAVI, papers with a study population of less than 100, unpublished Literature, gray Literature, and low-quality studies.

Study Selection and Data Extraction

The studies were selected through a two-step process. In the first phase, two reviewers (AD and DD) independently ran the search criteria in the databases and reviewed the titles and abstracts to determine eligibility. In the second phase, one reviewer (AD) obtained full-text articles from the initial screen and independently determined if articles were to be included. A third reviewer assessed the validity of the reasons for the excluded articles. Data were extracted from the final set of studies by the first reviewer (AD) and verified by the third reviewer. Extracted data included author name(s), publication year, location, study design, setting, population, size, intervention, method of PH assessment, criteria for PH diagnosis, outcome measures, and results.

Data Analysis

We performed a qualitative analysis of the included studies. Given the heterogeneity of the study and patient population, we could not perform a meta-analysis.

Quality Assessment

The quality of observational studies was assessed using the Newcastle-Ottawa scale, a quality evaluation tool for nonrandomized studies that evaluate studies based on selection, comparability, and outcome [23]. Any study scoring 3-4, 1-2, and 2-3 in the selection, comparability, and outcome/exposure domains were considered “good quality.” A study scoring 2, 1-2, and 2-3 in the selection in these domains were considered “good quality.” A study scoring 2, 1-2, and 2-3 in these domains were of “fair quality.” In all other cases, the study was considered “poor quality.” We attempted to minimize study selection bias by having predefined inclusion and exclusion criteria, dual review, and documentation of reasons for excluding studies. The quality appraisal of the studies has been described in Table 1.

**Table 1:** Quality appraisal of observational studies using the Newcastle-Ottawa tool

Study	Representative of Exposed Cohort	Non-Exposed Cohort	Assessment of Exposure	Outcome of interest not present at the start of the study	Comparability	Assessment	Follow up	Adequacy of f/u	Total
O'Sullivan et al. [1]	1	0	1	1	1	1	1		6
Sultan et al. [12]	1	1	1	1			1		5
Alushi et al. [15]	1	1	1	1	1	1	1		7
Sinning et al. [16]	1	1	1	1	1	1			6
Masri et al. [17]	1	1	1	1	1	1	1	1	8
Schewel et al. [18]	1	1	1	1	1	1	1		7
Barbash et al. [21]	1	1	1	1	1	1	1		7
Testa et al. [24]	1	1	1	1	1	1	1		7
Nijenhuis et al. [25]	1	1	1	1	1		1	1	7
Kleczynski et al. [26]	1	1	1		1		1		5
Keymel et al. [27]	1	1	1	1	1	1	1		7
Mujeeb et al. [28]	1	1	1	1	1				5

One study was an RCT that had a low to moderate risk for bias. The two meta-analyses were of moderate quality.

Results

Literature Search

**Figure 1:**
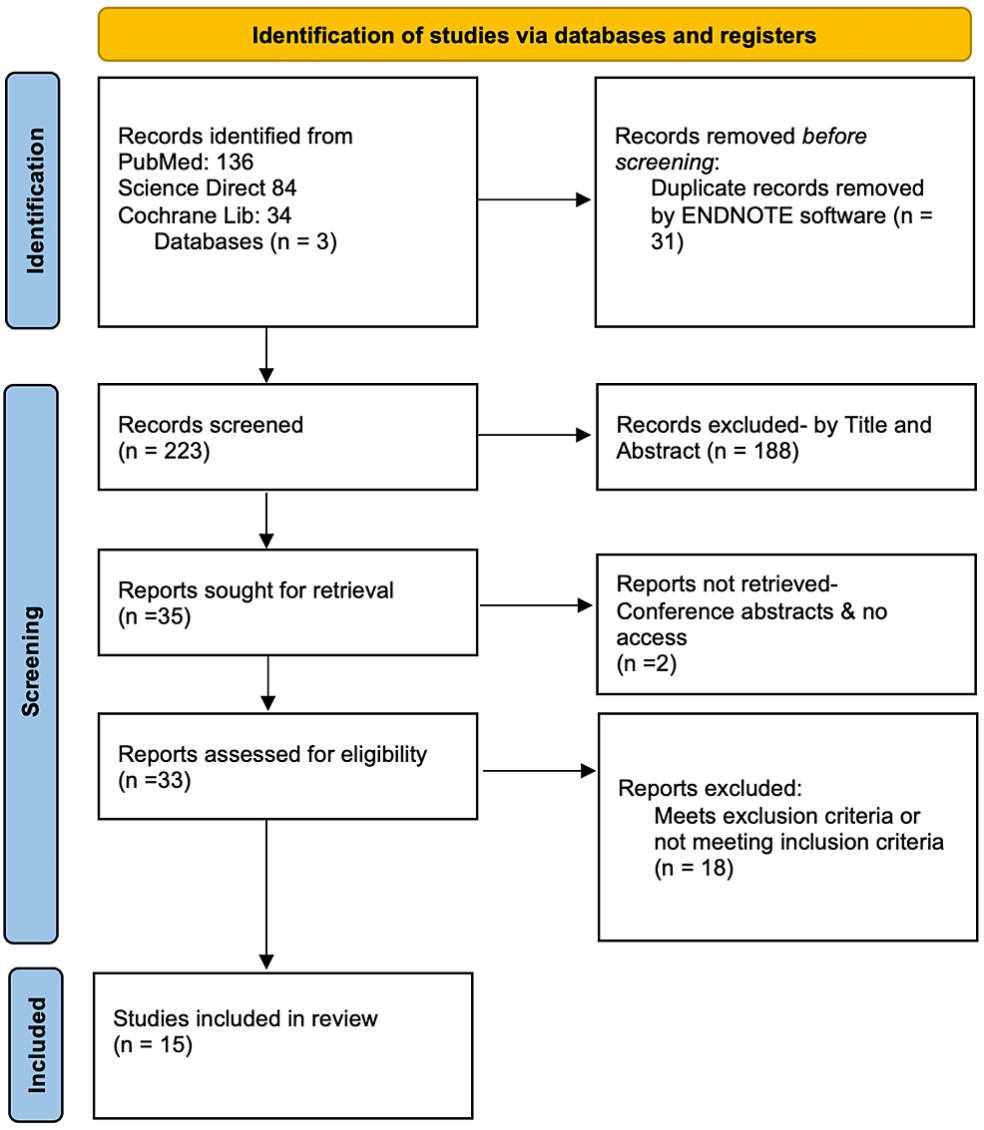
PRISMA Flow Diagram for Systematic Reviews- Study Inclusion Protocol

A total of 139 unique articles were identified and screened. Of the 33 full-text articles reviewed, 18 articles, including duplicates, were excluded for reasons listed in Figure 1. Fifteen articles fulfilled the selection criteria and were included in this review.

Study Characteristics 

Individual study characteristics are listed in Table 2. All the studies assessed this participant for outcome post-TAVI among patients with PH [1,11,12,15-18,21,24-30]. Half of the studies were conducted in the United States, the other half were from Europe and one study was from China. The study design included two meta-analyses, one randomized control trial, and 11 retrospective cohort observational studies. The studies involved a total of approximately 30,000 patients. The average population age was around 80 years or more. The studies included patients with severe AS undergoing TAVI. PH was diagnosed either with right heart catheterization (RHC) or echocardiography.

**Table 2:** Individual Study Characteristics TAVI- Transcatheter aortic valve implantation, PH- Pulmonary Hypertension, AS- Aortic stenosis, NYHA- New York Heart Association, AVA-Aortic valve area, RHC- Right Heart Catheterization, RCT- Randomized Controlled Trial, Ipc-PH: Isolated post-capillary Pulmonary Hypertension, Cpc-PH: Combined pre and post-capillary Pulmonary Hypertension, Pc-PH: Precapillary Pulmonary Hypertension.

Study Name	Year	Type of Study	Population	Country	Number of Patients	Percentage of population with PH	Comparison group	Age (years)	Proportion of Male (%)
Sinning et al. [16]	2014	Prospective Study	Patient with severe AS undergoing TAVI	Germany, United Kingdoms	353	71%	No PH vs mild to moderate PH vs Severe PH	81.1±6.6	52.6
Lindman et al. [29]	2015	RCT with continued access registry	Patients were symptomatic (NYHA) functional class ≥2 and had severe (AS) with an (AVA) <0.8 cm2 (or indexed AVA <0.5 cm2/m2) and either resting or inducible mean gradient >40 mm Hg or peak jet velocity >4 m/s	USA	2180	64%	No PH vs. Mild vs. Severe	85±7 vs. 84±7 vs. 83±8	47.00%
O'Sullivan et al. [1]	2015	Prospective cohort	Severe symptomatic AS (index AVA<0.6 cm2/m2) undergoing a preprocedural RHC were considered for inclusion	Switzerland	433	75%	No PH vs. PH and PH was dichotomized into Post Cap PH vs. Pre-Cap PH. Post Cap PH was again dichotomized into Isolated Post Cap PH and Combined PH	82.4±5.3	44%
Barbash et al. [21]	2015	Observational	Symptomatic severe AS who underwent TAVI from 2007 to 2013	USA	415	59%	No/mild PH vs Moderate/severe PH	84±8	47%
Testa et al. [24]	2016	Observational	Patient with Severe AS undergoing TAVI	Italy	999	22%	No PH vs. Moderate PH vs. Severe Ph	Group 1- 82±5 Group 2 - 81±7 Group 3 - 78±6	46%
Nijenhuis et al. [25]	2016	Observational	Patient undergoing TAVI	Netherland	687	59%	Low vs. Moderate vs. High probability of PH based on TRV on TTE	80.8±6.8	43.70%
Kleczynski et al. [26]	2017	Observational	Severe AS with Contraindication to SAVR and undergoing TAVI	Poland	148	43.9%	PH vs no PH	81.2±5.9	37.80%
Masri et al. [17]	2018	Retrospective	Patient undergoing TAVI	United States	407	67	No PH vs. Persistent PH Post TAVI	82±7	50
Schewel et al. [18]	2019	Retrospective	Symptomatic severe AS undergoing TAVI	Germany	1400	53		81.5±6.8	46.3
Alushi et al. [15]	2019	Retrospective	Patient with Severe AS undergoing TAVI	Germany, Switzerland, and the United Kingdom. Multicenter	617	49	Reversible PH post-TAVI vs. Residual PH post-TAVI	81±7	49
Keymel et al. [27]	2019	Retrospective	Patient with Severe AS undergoing TAVI	Germany	125	58.4	AS with PH and AS without PH	AS + PH vs. AS − PH: 78.9 6.3 vs. 78.0 5.9	67%
Sultan et al. [12]	2020	Retrospective	Patient undergoing TAVI	United States	614	67.3	Pc-PH, Ipc-PH vs. No PH, Cpc-PH vs. no PH	82.4±7.8	51%
Mujeeb et al. [28]	2021	Retrospective	National Database of patients who underwent TAVI	United States	13085	12.7	PH with AS vs. No PH with AS	80.9±8.5	48%
Tang et al. [30]	2016	Meta-analysis	TAVI patient diagnosed with PH	China	9204	NA	PH with AS vs. no PH with AS	-	-
Kokkinidis et al. [11]	2018	Meta-analysis	Severe AS undergoing TAVI	United States	17634	-	-	-	-

Association of Pulmonary Hypertension with Outcomes

Table 3 lists the results from various studies for short-term and long-term cardiac and all-cause mortality. Out of 15 studies, 12 studies recorded all-cause mortality in the hospital or at 30 days, out of which eight studies said that PH increases the 30 days of all-cause mortality or in-hospital mortality [11, 15, 18, 21, 24-26, 30]. Eight studies reported 30 days of cardiac mortality, and five of those studies showed significant 30 days of cardiac mortality associated with pulmonary hypertension. For example, Lindman et al. showed that PH was associated with increased one-year all-cause mortality [29]. Tang et al. showed that the pooled Odds Ratio (OR) for overall 30-day mortality was (OR-1.52) [30]. Nijenhuis et al., Kokkinidis et al., and Alushi et al. also supported the above findings [11,15,25].

**Table 3:** Results from included studies. TTE= Transthoracic echocardiography, TRV=Tricuspid Regurgitant Velocity, AS=Aortic Stenosis, PH=Pulmonary Hypertension, P< 0.05 is a statistically significant outcome, STS score=Society of Thoracic Surgeons Score, EuroScore=European System for Cardiac Operative Risk Evaluation, OR=odds ratio, HR= hazard ratio, PASP= Pulmonary artery systolic pressure, RR= Relative Risk, Pc-PH= Precapillary Pulmonary Hypertension, Ipc-PH= isolated postcapillary pulmonary hypertension and Cpc-PH= Combined pre and postcapillary pulmonary hypertension.

Study Name	Comparison group	30 days Cardiac Mortality	30 days all-cause mortality	Late Cardiac Mortality	Late all-cause mortality	Scoring system Used
Sinning et al. [16]	No PH vs Mild-moderate PH vs Severe PH	NA	(2%) vs (6.8%) vs (13.2%), P< 0.01	NA	(13.9%) vs (27.3%) vs (48.4%), p< 0.001	Logistic EuroScore & STS score
Lindman et al. [29]	No PH vs Mild PH vs Severe PH	(2.8%) vs (3.7%) vs (5.4%) p~0.051	p> 0.05	Death (cardiac) - (6.3%) vs (9.7%) vs (12.7%) <0.001	Death (all-cause) 144 (18.6%) vs 189 (22.7%) vs 138 (25.0%), p< 0.01	STS score
O'Sullivan et al. [1]	No PH vs PH- Ipc-PH vs Pcp-PH vs Cpc-PH	No statistical Significance	p> 0.05	Combined PH HR- 3.81 (1 year) p< 0.05	Combined PH HR- 3.28 (1 year), p<0.05	STS score & Logistic EUROscore
Barbash et al. [21]	No/mild PH vs Moderate/severe PH	No statistical Significance	14.5%, p< 0.02	NA	30.8% (1 year)	STS score & Logistic EUROscore
Testa et al. [24]	No PH vs. Moderate PH vs. Severe PH	No statistical Significance	p> 0.05	No PH vs Moderate PH/ Severe PH 8 vs 10 vs 15 (p < 0.05)	15% vs 20% vs 26% ( p <0.005)	STS score & Logistic EUROscore
Nijenhuis et al. [25]	Low vs Moderate vs High probability of PH based on TRV on TTE	5.9% vs. 5.3% vs. 11.9%, p = 0.04	5.9% vs. 6.1% vs. 18.9%, p < 0.01	20.1% vs. 23.4% vs. 33.8%, p < 0.01 (2 years)	26.8% vs. 28.7% vs. 47.1%, p < 0.01 (2 years)	STS score & Logistic EUROscore
Kleczynski et al. [26]	PH vs. no PH (TRV- Clinical parameters)	NA	NA	NA	OR 2.26 for a median follow-up of 13.3 months, p< 0.05	Logistic EUROscore
Masri et al. [17]	No PH vs. Persistent PH Post TAVI	NA	NA	NA	HR= 1.82, persistent PH at 1 month, p< 0.05	STS score
Schewel et al. [18]	No PH vs Pc-PH vs Ipc-PH vs Cpc-PH	NA	p < 0.0083 for no PH vs. Pc-PH. p < 0.0083 for no PH vs. Ipc-PH	1 year & 4-year mortality Pc-PH and Ipc-PH (p < 0.001) vs no PH and 4 years mortality Cpc-PH vs no PH p< 0.007	1 year and 4- year Pc-PH and Ipc-PH (p < 0.001) vs no PH, 4 years: Cpc-PH vs no PH p< 0.002	Logistic EUROscore
Alushi et al. [15]	Reversible PH vs Residual PH	p= 0.003	HR 4.41, p < 0.001	1 year: HR 2.84, p = 0.02, 5.9 years HR= 2.60, p= 0.002	1 year: HR= 3.69, P < 0.001, 5.9 years: HR= 2.80, p < 0.01	Logistic EUROscore
Keymel et al. [27]	AS with PH and AS without PH	NA	24.4 vs 3.8 p= 0.002	NA	NA	STS Score
Sultan et al. [12]	Pcp-PH, Ipc-PH vs No PH, Cpc-PH vs no PH	NA	NA	NA	Cpc-PH vs no PH [HR] 1.56, 95% CI p =0.025	STS Score
Mujeeb et al. [28]	PH vs No PH	NA	4.40% (in hospital) P< 0.001	NA	NA	NA
Tang et al. [30]	PH vs no PH	OR 1.53, p < 0.05	OR-1.52 p< 0.05	OR 1.56 (1 year), p< 0.05	OR 1.39 (1 year) OR 2.00 (2 year)	NA
Kokkinidis et al. [11]		Pooled RR 1.41, p< 0.05	HR 0.95 Pooled RR 1.71 p< 0.05	PASP > 60 subgroup, HR 1.8, p< 0.05	PASP > 60 mm Hg Subgroup, HR 1.56, p<0.05	NA

Twelve studies studied late all-cause mortality, concluding that PH was an independent risk factor for late all-cause mortality. Some studies also mentioned that certain subgroups were more likely associated with late all-cause mortality, such as Lindman et al. who showed that both moderate and severe PH was associated with increased mortality [29]. O'Sullivan et al. stated that Cpc-PH was associated with a Hazard Ratio (HR) of 3.28 at one year [1]. Barbash et al. supported the above findings with increased mortality among moderate and severe PH populations [21]. Tang et al. showed concomitant PH has OR-1.39 at one year and OR- 2 at two years in the TAVI population [30]. Testa et al. showed that severe PH was significantly associated with late all-cause mortality [24]. Nijenhuis et al. and a similar study showed that a high probability of pulmonary hypertension was associated with increased all-cause mortality [25,26]. Kokkinidis et al., in their meta-analysis, observed that PASP > 60 was associated with high all-cause mortality post-TAVI [11]. Masri et al. studied that persistent PH at one month was associated with a HR of 1.8 of late cardiac mortality following TAVI [17]. Schewel et al. showed that pre-capillary PH and Ipc-PH were associated with all-cause mortality at one year and four years and that the Cpc-PH was associated with all-cause mortality only at four years [18]. Alushi et al. observed that the residual PH post-TAVI is associated with increased one-year and late all-cause mortality [15], and Sultan et al. found that the combined PH was associated with an HR of 1.56 [12]. 

Eight studies evaluated that late-cardiac mortality was significantly associated with PH. Lindman et al. studied that severe PH is associated with increased late-cardiac mortality [29]. O'Sullivan et al. state that Cpc-PH was associated with an HR- 3.28 at one year [1], and Tang et al. reported baseline PH was associated with increased one-year cardiovascular mortality [30]. Testa et al. showed a similar association with severe PH [24]. Kokkinidis et al. observed that PASP > 60 was associated with an HR 1.8 for late cardiac mortality post-TAVI [11]. Schewel et al. stated that pre-capillary PH and Ipc-PH were associated with late cardiac mortality at one-year and four years; he also stated that Cpc-PH was associated with cardiac mortality at four years [18]. Residual PH post-procedure was associated with increased one year and at maximal follow-up of 5.9 years in a study by Alushi et al. [15].

Decrease in PASP or PH on Outcomes

Seven studies mentioned that there was a significant improvement in PASP after TAVI. O'Sullivan et al. reported improvement in PASP in Ipc-PH and Cpc-PH groups [1]. Barbash et al. said there was no significant difference in mortality at 30 days and one year in patients with decreased PASP post-TAVI [21]. Testa et al., in their observational study, mentioned that the decrease in PASP amongst the moderate and severe PH group compared to the mild PH group had significantly decreased atrial fibrillation (AF), severe mitral regurgitation (MR), and left ventricular ejection fraction (LVEF) < 30% [24]. Masri et al. could not prove the statistical association between mortality benefits and improved PASP post-TAVI [17]. In contrast, Schewel et al. demonstrated survival benefits among the Ipc-PH group in their study. Still, the decreased PASP in pre-capillary, and Cpc-PH did not show any survival benefit [18]. Alushi et al. compared residual PH vs. reduction in PH post-TAVI. They concluded that Residual PH post-TAVI was associated with high all-cause and cardiovascular mortality at 30 days, one year, and maximal follow-up of 5.9 years [15]. One prospective study demonstrated a decrease in PASP was significant after TAVI and persistent PASP or new onset severe PH defined as PASP > 60 mm Hg post-TAVI is associated with significant long-term mortality [16].

Discussion

PH can be commonly seen in patients with AS. Studies show that the prevalence of PH in patients undergoing TAVI can range from (12.7% to 75%) [24-30]. But a national estimate of 103,245 TAVI hospitalizations shows 12.7% had a diagnosis of PH [28]. The most common cause of PH in patients with AS is Ipc-PH due to back pressure changes from the left heart to the pulmonary vasculature. In a minority of the cases of AS undergoing TAVI, patients have Pc-PH or cpc-PH [1,12]. Therefore, we did a qualitative analysis of the studies looking for the prognostic implication of PH on patients undergoing TAVI. We found that pre-TAVI PH was significantly associated with short-term and long-term all-cause mortality and cardiovascular mortality. TAVI is associated with a significant decrease in Pre-TAVI PH, we noticed that post-TAVI persistent PH was associated with increased all-cause and cardiovascular mortality. This effect was most noticed in patients with severe PH post-TAVI.

Definition of PH

The prevalence of PH in the studies selected for this systematic review depended on the diagnostic method of PH and the cut-off value set by the authors. RHC is the gold standard for diagnosing PH and the type. PH can also be predicted non-invasively with the help of echocardiography by using PASP and Tricuspid Regurgitant Velocity (TRV) with additional parameters according to European Society of Cardiology (ESC) guidelines [31]. Two studies were meta-analyses which mainly included studies that assessed PH through echocardiographic parameters [11,30]. Mujeeb et al. grouped PH based on pre-existing diagnoses from the national inpatient database [28]. Six studies included in our systematic review used RHC to assess PH with a cut-off value of mean Pulmonary artery Pressure (mPAP) 25 mm Hg [1,12,17,18,27,29]. Five studies used echocardiographic parameters to suspect PH [15, 21, 24-26]. Out of those, three studies used PASP with different cut-off values to stratify PH. For example, Barbash et al. used the cut-off of 50 mm Hg for PASP to divide the study population into no/mild PH vs. moderate/severe PH [21]. Testa et al. divided their study population into three groups: PASP < 40 mm Hg- no PH, PASP 40-60 mm Hg- moderate PH, and PASP > 60 mm Hg- severe PH [24]. Alushi et al. defined PH as normal (PASP < 34 mm Hg), low to moderate (PASP >34 and <46 mm Hg), and severe (PASP >46 mm Hg) [15]. In contrast, a couple of them used TRV to suspect PH, using TRV < 2.8 m/s as the cut-off [25,26]. Table 4 shows the definition of PH used by studies in this analysis.

**Table 4:** Diagnostic methods and definition of pulmonary hypertension PASP-Pulmonary Artery Systolic Pressure, TAVI-Transcatheter Aortic Valve Implantation, mPAP-mean Pulmonary Artery Pressure, RHC-Right Heart Catheterization, TTE-Transthoracic Echocardiography, PH-Pulmonary Hypertension, TRV-Tricuspic Regurgitant Velocity, mPCWP-mean Pulmonary Capillary Wedge Pressure, PVR-Pulmonary Vascular Resistance, LEVF-Left Ventricular Ejection Fraction, Pc-PH-Precapillary PH, Ipc-PH-isolated post-capillary PH, and Cpc-PH-combined pre-capillary and post-capillary PH.

Study Name	Decrease in Pulmonary Hypertension Post Valve Implantation	Diagnostic Method for Pulmonary Hypertension
Sinning et al. [16]	Improvement in PASP was seen in about 83% of the population, persistent PASP > 60 mm Hg or new onset severe PH was associated with significant mortality post-TAVI.	TTE, PASP > 30 mm Hg
Lindman et al. [29]	Not Available	RHC, mPAP> 25
O'Sullivan et al. [1]	Significant improvements in right ventricular function and PASP were observed in both Icp-PH and Cpc-PH groups.	RHC, mPAP> 25
Barbash et al. [21]	The outcome was not different for the patients who experienced a decrease in PASP compared with those who did not, with 7.4% versus 8% for 30-day mortality (p~ 1) and 22.2% versus 28% for 1-year mortality (p ~ 0.56), respectively.	TTE, PASP > 50
Testa et al. [24]	After one month, the PASP decreased at least 15 mm Hg in 32% and 35% of the patients in groups two and three. Patients with a reduced PH one month after TAVI had a significantly lower rate of atrial fibrillation, severe MR, and severely depressed LVEF (<30%).	TTE, PASP> 40 mm hg
Nijenhuis et al. [25]	Pulmonary Artery pressure was not measured during follow-up.	TTE, “low” (TRV ≤2.8 m/s or not measurable without additional PH signs), “intermediate” (TRV ≤2.8 m/s or not measurable with additional PH signs, or TRV 2.9 to 3.4 m/s without additional PH signs), or “high” (TRV 2.9 to 3.4 m/s with additional PH signs, or TRV > 3.4 m/s)
Kleczynski et al. [26]	Not Applicable	TTE “low” (TRV ≤2.8 m/s or not measurable without additional PH signs), “intermediate” (TRV ≤2.8 m/s or not measurable with additional PH signs, or TRV 2.9 to 3.4 m/s without additional PH signs), or “high” (TRV 2.9 to 3.4 m/s with additional PH signs, or TRV >3.4 m/s)
Masri et al. [17]	When patients with PH at baseline were divided based on post-TAVI, TTE-derived PASP, there was no statistically significant difference in mortality between patients with no PH at baseline and those with improved PH; p = 0.557	TTE, PASP> 45 mm Hg
Schewel et al. [18]	There were no significant differences, either after one year or after four years of follow-up, in patients with Pc-PH and those with Cpc-PH. Interestingly, patients with Ipc-PH showed a significant overall survival benefit after 1 and 4 years in patients with reversible PH.	RHC, mPAP > 25 mm hg
Alushi et al. [15]	In patients with baseline PH (78%), a significant decrease in PH was observed in 49% at discharge and 59% one year after TAVI. 57% experienced reductions of their baseline PASPs with an overall mean reduction of 12 - 10 mm Hg (57.0±9.5 mm Hg vs. 45±11 mm Hg; p < 0.001) at discharge and 18-14mmHg (57.0±9.5mmHg vs. 39±13 mm Hg; p -0.001) and at 1 year.	TTE, PASP > 34 mm hg
Keymel et al. [27]	Not appplicable	RHC, mPAP > 25 mm hg
Sultan et al. [12]	Not applicable	PH defined as mPAP ≥ 25 mm Hg and was classified as pre-capillary PH (mPCWP ≤ 15 mm Hg), isolated postcapillary PH (Ipc-PH; mPCWP > 15 mmHg, PVR ≤ 3 Wood units), or combined pre- and postcapillary PH (Cpc-PH; mPCWP > 15 mmHg, PVR > 3 Wood Units).

Previous studies have shown that patients with PH undergoing SAVR and TAVI have high mortality. In our qualitative analysis, we were able to see a similar trend [1-4].

Thirty Days Mortality

Cardiovascular mortality: The meta-analysis by Tang et al. showed that the history of PH in the TAVI population increases 30 days of cardiac mortality, while it was not significant in meta-analysis by Kokkinidis et al. [11,30]. While Kokkinidis was done in the United States and included 22 observational studies, it only studied early and late mortality cases [11]. On the other hand, Tang et al. also studied the changes in PASP and the persistence of post-TAVI PH [25]. Out of 12 remaining studies, only six reported 30 days of cardiovascular mortality. Out of those, three studies showed it to be significantly associated with PH. All these three studies had different comparison groups. Alushi et al. compared reversible PH vs. residual PH by calculating PASP pre and post-TAVI [15]. On the other side, Nijenhuis et al. used echocardiographic TRV along with clinical variables to divide the TAVI population into low, intermediate, and high probabilities of PH [25]. Lindman et al. used the gold standard method to diagnose PH and divided their study into no PH vs. mild PH vs moderate and severe PH [29]. The other three studies, which reported 30 days of cardiovascular mortality but did not find it significantly associated with PH in the TAVI population, had fewer patients studied. Therefore, we think they were not able to reach statistical significance. AF was present in almost 50% of the patient population and can be correlated to the development of Ipc-PH due to retrograde changes and which in turn can cause left atrial enlargement.

All-cause mortality: Both the meta-analysis included in our qualitative synthesis found increased all-cause mortality post-TAVI. While Kokkinidis et al. just expanded and validated the meta-analysis by Tang et al., they also analyzed subgroups based on the PASP cut-off for short-term all-cause mortality. Still, it did not come to statistical significance [11,25]. The pooled HR only included five studies and was insignificant, but the RR was significant with moderate heterogeneity [11]. Tang et al. used OR to justify increased short-term all-cause mortality [25]. Five Observational Studies showed increased all-cause mortality [18,21,26,28,30]. Mujeeb et al. only reported in-hospital mortality to be high as they used the national inpatient database [30]. Keymel et al. had to combine the all-cause mortality with the number of cardiopulmonary resuscitation (CPR) to reach statistical significance [28]. Schewel et al. could only conclude short-term mortality in patients with Ipc-PH and Pc-PH [18]. Barbash et al. and Nijenhuis et al. showed increased short-term mortality [21,26]. Some studies attributed short-term mortality to the development of acute kidney injury (AKI) post-TAVI, and its prevalence was noted to be (~30% to ~50%) in the included studies. It can also be attributed to prolonged Intensive Care Unit/hospital stays. These findings suggest that there is a need for pre-TAVI management of PH.

Mortality After One Year

Cardiovascular mortality: Two meta-analysis in our study and six other observational studies found PH to be significantly associated with late cardiac mortality. Kokkinidis et al. stated that the PASP > 60 subgroups were at higher risk; only six studies had reported late cardiac mortality in their study [11]. Subgroup analysis of the other metanalysis did not show any statistical significance in increased cardiovascular mortality [30]. While Alushi et al. studied that residual PH post-TAVI was associated with late cardiovascular mortality at one year and a maximum follow-up of 5.9 years [15]. Schewel et al. demonstrated that PH had increased mortality in subgroups with Ipc-PH and pre-capillary PH at one year and four years, but Cpc-PH only significantly increased mortality at four years; they attributed it to a smaller subgroup population [18]. Nijenhuis et al. studied outcomes at two years, high probability of PH was strongly associated with cardiovascular mortality [25]. Testa et al. also reiterated the same thing. Still, they also analyzed the change in PH post-TAVI and reported that severe PH at baseline and persistence of the same post-TAVI is associated with higher mortality [24]. O'Sullivan et al. mentioned that Cpc-PH was significantly associated with the outcome, but they only included the patient who had right heart catheterization in their study, and it might have overestimated their study results; therefore, their results might not be generalizable [1]. The RCTs in our review supported the above studies. Still, they did not have the baseline and post-TAVI echocardiographic parameters. Lindman et al. could not comment on the change in PH post-TAVI and how it affects cardiovascular mortality [29]. Overall, studies analyzing cardiovascular mortality are limited; the ones in the review suggested increased cardiovascular mortality with baseline PH, but data on changes in PASP post-TAVI and its effect on cardiovascular mortality are limited.

All-cause mortality: A meta-analysis from China and the US supported the findings of the observational studies included in our analysis [11,30]. The RCT by Lindman et al. and a retrospective study recommended that along with the objective finding of PH, clinical findings were also important in determining the mortality and should be considered while making decisions for doing a TAVI [25, 29]. While Kleczynski et al. demonstrated increased all-cause mortality by stratifying PH using TRV only, they demonstrated no effect on Quality of Life in patients with PH; more studies with a larger cohort are needed to confirm the same [25]. Studies by O'Sullivan and Sultan et al. were only able to demonstrate the correlation of high mortality with Cpc-PH [1,12]. One of them states that the Cpc-PH had severe PH at baseline with a diastolic pressure difference (DPD) > 7 mm Hg, which is known to cause advanced pulmonary vascular remodeling [12]. Similar results were also generated by Schewel et al. as they found cpc-PH at four years and Pcp-PH and Icp-PH at both one year and four years to be significant [18]. Both were biased as they were not consecutive patient series and may not be generalizable to the population. Barbash et al. did a post-hoc analysis and found similar results with severe PH at baseline [21]. Three observational studies reiterated the same thing that persistent PH post-TAVI is associated with increased mortality but had a different definition of persistent PH and its correlation; for example, Testa et al. found a correlation with severe PH (PASP > 60) [24], Masri et al. defined moderate persistent PH post-TAVI (PASP > 45) as an association [17] and as Alushi et al. correlated residual PH or no change in PH or worsening of PH post-TAVI as a predictor of mortality [15]. Overall, all the studies conclude that baseline PH and persistent PH post-TAVI are independent risk factors for late all-cause mortality.

Effect of Decrease in PH

A decrease in PASP after the TAVI has been reported in very few studies, and some of them are included in our review here. As we know, PH can be categorized as Pc-PH, Icp-PH, or Cpc-PH. The studies included in our analysis observed significant improvement in PASP in the Ipc-PH and Cpc-PH groups. The underlying mechanism can explain that: Severe AS leads to increased resistance in the left ventricular outflow, which in turn causes hypertrophy of the left ventricle and progressive diastolic dysfunction. This change causes elevated Left ventricular end-diastolic pressure and, in turn, elevated left atrial filling pressure. All of this causes retrograde changes in the pulmonary veins and, in turn, involves pulmonary arteries. It can be hypothesized that initially severe AS can cause Ipc-PH. Still, as it progresses, it causes intrinsic pulmonary artery changes and eventually involves the right ventricle causing Cpc-PH. Therefore, the most improvement is seen in the patient in the Ipc-PH group.

O'Sullivan, in his study, mentioned that Pc-PH had worse outcomes, and they noticed a significant improvement in PASP and right ventricular function in the Ipc-group [1]. However, they did not study the mortality benefits of decreasing PASP. Barbash et al., in their study, could not prove a post-TAVI decrease in PH and its impact on mortality benefits; we think it is related to their definition of a decrease in PASP post-TAVI as they defined improvement in TAVI as a decrease in PASP ≥ 10 mm Hg [21]. Testa et al. defined a decrease in PASP as at least 15 mm Hg in their study; they were not able to prove mortality benefits; however, they concluded that a decrease in PASP ≥ 15 mm Hg was related to the absence of AF, severe MR and severely decreased LEVF [24]. Masri et al. expanded the study done by Testa et al. They concluded that residual moderate or higher PH was associated with increased all-cause mortality compared to Improved PH but again failed to demonstrate the mortality benefit of improved PH post-TAVI compared to no PH group [17].

Schewel et al. showed a significant survival benefit in patients with Ipc-PH who had a decrease in pulmonary systolic pressure to normal values post-TAVI, which comprised almost 80% of the study population. At one year and four years, the other subgroups in the study had a decrease in PASP but had no survival benefits, which can be explained by the reversal of the underlying PH mechanism in the Ipc-PH subgroup [18]. Alushi et al. concluded that reversible PH or reduction in PASP had lower short and long-term all-cause and cardiovascular mortality due to concomitant reduction in severe MR, Tricuspid regurgitation, and Right Ventricular dysfunction [15]. They also mentioned LVEF > 40%, logistic EuroSCORE < 25%, the presence of severe baseline PH, and the absence of moderate to severe TR as an independent factor for reversible PH. The persistence of PH can be explained by irreversible right ventricular (RV) dysfunction secondary to long-standing elevated PASP, which in turn carries higher mortality. Very few studies here could justify that improvement in PH carries mortality benefits. Overall, PH is a risk factor for TAVI, but it's only included in EuroScore scoring systems. Our studies reported EuroScore and STS scores depending upon the researcher to risk-stratify patients before TAVI.

Therefore, the development of irreversible RV dysfunction poses higher mortality. Therefore, interventions should be made to prevent the progression, which can be done by screening and performing TAVI in the early stage of AS or medically managing the pre-capillary component of PH in the pre-TAVI phase. Table 4 describes the effect of Post TAVI reduction on PH.

Limitations

This was a systematic review, with the majority being observational studies. Therefore, our results should be interpreted in context to that and the biases about observational studies as most of them had a retrospective design. There were only two meta-analyses and one RCT. Some of them were not specifically designed to study PH and its outcomes and there is a lot of heterogeneity. We could not get full-text articles for a couple of studies as they were either paid, not accessible or conference abstracts for which full text was not available. The studies were not uniform in their definition of PH and the method of diagnosing PH, which can underestimate or overestimate study results. Some of the studies lacked the proper definition of cardiovascular mortality.

## Conclusions

Baseline PH and persistence of PH post-TAVI are strongly associated with all-cause and cardiac mortality. RCT are needed to study the cardiovascular effects as very few studies reported cardiovascular mortality. There is a need to form a uniform definition with the standard diagnostic method for PH in patients undergoing TAVI and include it in the scoring system along with clinical parameters. Few studies have shown that a decrease in post-TAVI PH carries mortality benefits. Therefore, efforts should be made to identify mechanisms of persistent PH post-TAVI and whether interventions to reduce PH pre-TAVI will have any clinical implications or not by conducting RCT.

## Appendices

**Table 5:** Pubmed search strategy

Search number	Query	Sort By	Filters	Search Details	Results	Time
5	("Transcatheter Aortic Valve Replacement"[Mesh]OR "TAVR" OR "TAVI" OR "Transcutaneous aortic valve replacement" OR "transcutaneous aortic valve implantation") AND ("Hypertension, Pulmonary"[Mesh] OR "Pulmonary Arterial Hypertension"[Mesh] OR "PH" OR "Pulmonary Hypertension" OR "PAH")		Full text, Humans	(("Transcatheter Aortic Valve Replacement"[MeSH Terms] OR "TAVR"[All Fields] OR "TAVI"[All Fields] OR "Transcutaneous aortic valve replacement"[All Fields] OR "transcutaneous aortic valve implantation"[All Fields]) AND ("hypertension, pulmonary"[MeSH Terms] OR "Pulmonary Arterial Hypertension"[MeSH Terms] OR "PH"[All Fields] OR "Pulmonary Hypertension"[All Fields] OR "PAH"[All Fields])) AND ((fft[Filter]) AND (humans[Filter]))	136	17:55:04
4	("Transcatheter Aortic Valve Replacement"[Mesh]OR "TAVR" OR "TAVI" OR "Transcutaneous aortic valve replacement" OR "transcutaneous aortic valve implantation") AND ("Hypertension, Pulmonary"[Mesh] OR "Pulmonary Arterial Hypertension"[Mesh] OR "PH" OR "Pulmonary Hypertension" OR "PAH")		Humans	(("Transcatheter Aortic Valve Replacement"[MeSH Terms] OR "TAVR"[All Fields] OR "TAVI"[All Fields] OR "Transcutaneous aortic valve replacement"[All Fields] OR "transcutaneous aortic valve implantation"[All Fields]) AND ("hypertension, pulmonary"[MeSH Terms] OR "Pulmonary Arterial Hypertension"[MeSH Terms] OR "PH"[All Fields] OR "Pulmonary Hypertension"[All Fields] OR "PAH"[All Fields])) AND (humans[Filter])	140	17:54:56
3	("Transcatheter Aortic Valve Replacement"[Mesh]OR "TAVR" OR "TAVI" OR "Transcutaneous aortic valve replacement" OR "transcutaneous aortic valve implantation") AND ("Hypertension, Pulmonary"[Mesh] OR "Pulmonary Arterial Hypertension"[Mesh] OR "PH" OR "Pulmonary Hypertension" OR "PAH")			("Transcatheter Aortic Valve Replacement"[MeSH Terms] OR "TAVR"[All Fields] OR "TAVI"[All Fields] OR "Transcutaneous aortic valve replacement"[All Fields] OR "transcutaneous aortic valve implantation"[All Fields]) AND ("hypertension, pulmonary"[MeSH Terms] OR "Pulmonary Arterial Hypertension"[MeSH Terms] OR "PH"[All Fields] OR "Pulmonary Hypertension"[All Fields] OR "PAH"[All Fields])	173	17:54:47
2	"Hypertension, Pulmonary"[Mesh] OR "Pulmonary Arterial Hypertension"[Mesh] OR "PH" OR "Pulmonary Hypertension" OR "PAH"			"hypertension, pulmonary"[MeSH Terms] OR "Pulmonary Arterial Hypertension"[MeSH Terms] OR "PH"[All Fields] OR "Pulmonary Hypertension"[All Fields] OR "PAH"[All Fields]	611,411	17:54:33
1	"Transcatheter Aortic Valve Replacement"[Mesh]OR "TAVR" OR "TAVI" OR "Transcutaneous aortic valve replacement" OR "transcutaneous aortic valve implantation"			"Transcatheter Aortic Valve Replacement"[MeSH Terms] OR "TAVR"[All Fields] OR "TAVI"[All Fields] OR "Transcutaneous aortic valve replacement"[All Fields] OR "transcutaneous aortic valve implantation"[All Fields]	13,966	17:54:05
